# Prevalence of chronic kidney disease in people with severe mental illness: A systematic review and meta-analysis

**DOI:** 10.1192/bjp.2026.10659

**Published:** 2026-05-26

**Authors:** Claire Carswell, Rebecca Nisbet, Jakiah Khan, Zaahidah Patel, Ammaarah Husain, Kate Bramham, Joseph Chilcot, Rowena Jacobs, David Osborn, Najma Siddiqi

**Affiliations:** 1 Department of Health Sciences, University of York, York, United Kingdom; 2 Hull York Medical School, York, Unite d Kingdom; 3 Faculty of Health and Life Sciences, De Montfort University, Leicester, United Kingdom; 4 Department of Renal Sciences, King’s College London, London, United Kingdom; 5 Department of Psychology, Institute of Psychiatry, Psychology and Neuroscience, King’s College London, London, United Kingdom; 6 Centre for Health Economics, University of York, York, United Kingdom; 7 Division of Psychiatry, University College London, London, United Kingdom; 8 Bradford District Care NHS Foundation Trust, Bradford, United Kingdom

## Abstract

**Background:**

People with severe mental illness (SMI) are more likely to develop long-term physical health conditions compared to people without SMI. This contributes to an inequality in life expectancy. Chronic kidney disease (CKD) is a growing global health concern set to be the 5th leading cause of life-years lost by 2040. People with SMI may have a higher risk of CKD, however there is limited research exploring the relationship between CKD and SMI.

**Aims:**

This review aimed to examine the prevalence, incidence and risk of CKD among people with SMI.

**Methods:**

We searched Medline, Embase, PsycINFO, CINAHL, Scopus and Web of Science for epidemiological research reporting the prevalence of CKD (of any stage according to Kidney Disease Improving Global Outcomes (KDIGO) guidelines) among people with SMI. Records were imported into Covidence and screened by two reviewers. Meta-analyses were conducted using random effects models to examine the prevalence, incidence and risk of CKD among people with SMI.

**Results:**

Forty-eight studies were included in the review. The pooled prevalence of CKD was 8% in studies of people with SMI (95% CI= 5%, 18%) and was highest in studies focused only on participants with bipolar disorder (0.15 (0.06, 0.26)). The pooled incidence rate of CKD was 26.83 cases (95% CI = 18.66,38.58) per 1,000 person-years. People with SMI had significantly higher odds of CKD compared to people without SMI (OR = 2.33 (95% CI = 1.70, 3.21)).

**Conclusion:**

People with SMI are at a significantly higher risk of having CKD compared to people without SMI. While psychiatric medication and high rates of diabetes may play a role, the drivers of this inequality are under-researched.

## Introduction

Severe mental illnesses (SMI; conditions such as schizophrenia, schizoaffective disorder and bipolar disorder) are associated with a significant reduction in life-expectancy, known as the mortality gap.(1) On average, people with SMI die 15-20 years earlier than people who do not have SMI(1, 2). While a portion of this variation is attributable to suicide and accidental death, the main contributing factor to this inequality is the largely preventable poor physical health of people with SMI(1–3). This includes higher rates of long-term physical health conditions and poorer outcomes from those conditions (4–7).

CKD is a progressive condition that is often asymptomatic in the early stages and is characterised by a sustained reduction in kidney function, typically staged by estimated glomerular filtration rate (eGFR) (11). There are five stages of CKD according to the Kidney Disease Improving Global Outcomes (KDIGO) guidelines, and in the latest stage (Stage 5) kidney function is severely impaired, necessitating kidney replacement therapies including dialysis or renal transplantation (11). According to recent global health estimates, CKD affects approximately 843.6 million individuals worldwide, a figure that continues to rise due to population ageing, increasing rates of diabetes and hypertension, and other potentially modifiable risk factors (12). Projections indicate that by the year 2040, CKD will rank as the fifth leading cause of life-years lost globally, highlighting its growing impact on public health systems (13).

Cardiovascular death is the leading cause of mortality among individuals living with SMI, accounting for approximately 70% of all deaths in those diagnosed with bipolar disorder or schizophrenia(5). Epidemiological studies have shown that individuals with SMI have a significantly elevated risk of both sudden cardiac death and overall cardiovascular mortality (5, 8). Cardiovascular mortality is also the leading cause of death among people with chronic kidney disease (CKD) (9, 10). While the relationship between SMI and long-term physical health conditions such as cardiovascular disease and type 2 diabetes has been relatively well documented (5, 7, 14, 15), chronic kidney disease (CKD) has not attracted the same attention, despite available evidence suggesting that CKD may disproportionately impact people with SMI (16).

Several medications used in the treatment of SMI can increase the risk of CKD (17). Lithium, a highly effective mood stabiliser used in the management of bipolar disorder and schizoaffective disorder, is associated with a range of renal side effects (18). This includes nephrogenic diabetes insipidus, tubulointerstitial nephritis, and long-term decline in glomerular function (17). Acute kidney injury (AKI) can also occur in cases of lithium toxicity, when serum lithium levels are elevated outside the narrow therapeutic range (19). Antipsychotic medications, while not exhibiting the same renal side effects, are known to induce or exacerbate metabolic syndrome, a cluster of conditions including obesity, dyslipidaemia, insulin resistance, and hypertension, all of which are independently associated with increased risk of CKD (20).

Antipsychotic medications also contribute to the development of other conditions, such as type 2 diabetes mellitus and hypertension, which are well-established risk factors for CKD (21, 22). Additionally, health risk behaviours associated with CKD are more prevalent among people with SMI – including high rates of smoking, diets high in saturated fats, sugars, and sodium, and high levels of sedentary behaviour (23). Social determinants of health, such as poverty, housing insecurity, and reduced access to preventive health services, may exacerbate these risks and contribute to poorer health outcomes in this population, including a higher risk of CKD (24, 25).

Early identification and proactive management of CKD are critical to mitigate progression to kidney failure and reduce cardiovascular mortality risk (26, 27). Timely intervention, including identification of deteriorating eGFR and management of hypertension and diabetes, could slow CKD progression and improve survival outcomes (26, 28). To contribute to improved identification and appropriate care for people with co-existing SMI and CKD, the epidemiology of this co-morbidity, including its prevalence and incidence, needs to be better understood. To date, there has been no systematic review or meta-analysis on the risk of CKD among people with SMI (16). Therefore, we conducted a systematic review to describe the prevalence, incidence and risk of kidney disease among people with SMI.

## Objectives

This review had two overarching objectives:

- Estimate the prevalence and incidence of CKD among people with SMI.
- Compare the prevalence and incidence of CKD among people with SMI to those in the general population or those who do not have SMI.

## Methods

This systematic review protocol was prospectively published (29) and registered in the International Prospective Register of Systematic Reviews (PROSPERO) CRD42024527215 (30).

### Search strategy

We initially searched Medline, Embase, PsycINFO, CINAHL, Scopus and Web of Science from conception until June 2024, and re-ran the search in February 2025. We used search terms specific to the population (people with severe mental illness), outcome (chronic kidney disease) and study design (epidemiological studies). The full search strings for the electronic databases can be found in the Supplementary Materials. We also carried out forward and backwards citation searches to identify any relevant records not included in the database search and searched the archives of subject-specific journals.

### Study selection

Studies were included if they were epidemiological observational studies that reported the prevalence or incidence of CKD (any stage) in a population of adults with SMI. SMI was defined as any condition that can present with psychosis, including schizophrenia, schizoaffective disorder, bipolar disorder and depression with psychosis.

Studies were excluded if the denominator population did not consist of people with SMI, or a majority with SMI (over 50% of the denominator population). If studies did not report the proportion of people with SMI, they were also excluded. Studies were also excluded if they included participants who were under the age of 18 (unless data for participants over the age of 18 could be extracted and analysed separately).

Studies were also excluded if they only reported the prevalence or incidence of acute kidney injury (AKI) or if the study did not differentiate AKI from CKD.

### Data extraction (selection and coding)

Screening and identification of included records was conducted using Covidence. Title, abstract and full-text screening was completed by CC, RN, JK, AH and ZP, with two independent reviewers reviewing each of the records at both stages, and any disagreements resolved through discussion and consensus or, if needed, consultation with an independent author.

Data extraction started on the 05/09/2024. The data extraction table was piloted by CC and RN, and data were extracted for each article by two independent reviewers (CC, RN, JK, ZP or AH). The extracted data included author, year, publication type, aim of the study, country, World Bank designation, setting, study design, study duration, year(s) of data collection, sample size, participant demographics, SMI diagnostic tool, SMI diagnoses, psychiatric medication, data collection procedures, CKD stages and definition, prevalence of kidney disease, and incidence of kidney disease. Studies that included a comparison group of the general population or a population without SMI had additional data extracted on the demographics of comparator groups, the prevalence or incidence of CKD in the comparator group, and any risk, odds, or hazard ratios reported.

### Risk of bias

The risk of bias for each included study was assessed by two independent reviewers (CC, RN, JK, ZP or AH) using the appropriate JBI Critical Appraisal tools. Where discrepancies arose between scores or the choice of checklist, this was resolved through discussion and consensus, and if needed, referred to a third independent reviewer. Each study was scored out of the total number of items that applied to the study. There is no validated categorisation of quality within the JBI critical appraisal tools, so for this review studies with no items checked ‘no’ or ‘unclear’ were assessed as high quality, those with one item checked as ‘no’ or ‘unclear’ were assessed as moderate quality, and those with two or more items checked as ‘no’ or ‘unclear’ were assessed as low quality.

## Data analysis

### Narrative synthesis

The characteristics of the included studies were summarised and described to provide an overview of the evidence.

### Meta-analysis

Random effect models were used in the meta-analyses to account for the high level of heterogeneity. As per the protocol, clinically heterogeneous studies were excluded from the meta-analyses due to the lack of similarity of the patient cohorts (for example, studies that only reported the prevalence of CKD among people with SMI and a specific co-morbidity were excluded from the analysis). Studies were also excluded if their study design did not result in reporting prevalence or incidence in a way that would allow pooling. In the meta-analyses of ratios, studies were only included if the comparison was between the prevalence or incidence of CKD in people with SMI and people without SMI or the general population. Comparisons to other populations, such as people with other mental health conditions (such as anxiety or depression), or between different types of psychiatric drugs, were not pooled.

Five meta-analyses were conducted:

- The pooled prevalence of CKD among people with SMI.
- The pooled incidence risk of CKD among people with SMI.
- The pooled incidence rate of CKD among people with SMI, per 1000 person-years at risk.
- The pooled odds ratio of CKD among people with SMI compared to the general population.
- The pooled hazard ratio of CKD among people with SMI compared to the general population.

### Sensitivity analyses

Where possible, sensitivity analyses were conducted to determine whether the method of identifying CKD influenced the pooled result. The methods of identification of CKD were categorised into the following:

- Diagnostic code
- eGFR calculation
- eGFR calculation and albuminuria
- eGFR calculation and kidney biopsy
- Self-report

The sensitivity analysis involved removing the studies which used self-report, albuminuria or kidney biopsy from the meta-analyses where relevant (as some of the meta-analyses did not include studies which used these methods of CKD identification) and examined the impact on the overall results. Finally, we conducted a subgroup analysis to explore whether pooled prevalence estimates differed according to the use of diagnostic codes or eGFR calculations.

### Certainty of Evidence

We used the Grading of Recommendations, Assessment, Development and Evaluations (GRADE) approach to determine the certainty of the synthesised evidence. GRADE is predominantly used to assess the certainty of evidence on the effectiveness of interventions. While it has not been formally adapted for systematic reviews of prevalence or incidence, we used an approach recommended by Borges Migliavaca et al. (2020) (31), for applying GRADE to baseline risk or prognosis reviews.

## Results

**Figure 1:**
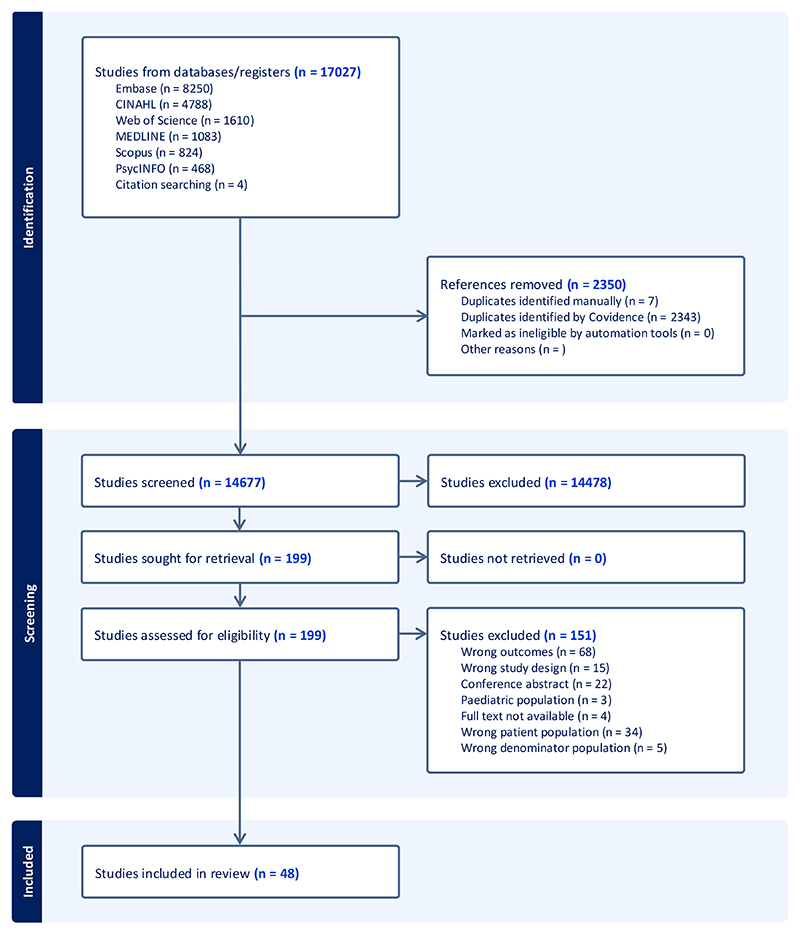
PRISMA Flow diagram

Figure 1 shows the Prisma flow diagram. Following the removal of duplicates (n=2350), 14677 records were screened at the title and abstract stage, with 199 full-text articles screened for eligibility. At this stage, 151 articles were excluded. Reasons for exclusion were reporting wrong outcomes (n=68), wrong patient population (n=34), and wrong study design (n=22). Records were also excluded if the full text was unavailable after contacting authors and requesting interlibrary loans, including studies only available as conference abstracts (n=22). In total, 48 studies were identified for inclusion in the review.

### Description of included studies

Table 1 shows the characteristics of the included studies. The sample size of participants with SMI in the included studies ranged from 61 to 848,058, with a mean of 27,379. Publication dates ranged from 2003 to 2025, with more than half published between 2020-2025. Most studies used a cohort design (n= 30, 62.5%) and were published in high-income countries (HICs) (n= 45, 94%). The most common countries reported were the UK (n=10, 20.8%), USA (n=7, 14.6%), Denmark (n=6, 12.5%) and Sweden (n=5, 10.4%). The majority of studies used population-level data (n=31, 64.6%), while 7 studies collected data in a community setting (14.6%) and 4 collected data in inpatient settings (8.3%).

In terms of the denominator population, 17 studies only collected data from people with bipolar disorder (35.4%), and 11 studies included only people with schizophrenia (22.9%). Six studies included people with bipolar disorder and schizoaffective disorder (12.5%), and the remaining studies collected data from a population with mixed SMI diagnoses (n=14, 29%). Thirteen studies collected data only from people who had been exposed to lithium (27.1%), and thirteen studies collected data from populations with mixed exposure to lithium (27.1%), where a proportion of people were prescribed lithium, while others were not. The remaining 22 studies (45.8%) did not report the proportion of the population prescribed lithium.

Studies reported the prevalence or incidence of CKD according to different stages. 27 studies (56.3%) used the catch-all term ‘chronic kidney disease’ without defining specific stages or eGFR thresholds to report the prevalence, and 13 studies (27.1%) reported stage 3 and above, typically defined as eGFR <60 ml/min/ml2. Most studies used medical records to identify CKD, either through diagnostic codes (n=20, 62.5%) or eGFR calculations based on data from medical records (n=14, 29.2%).

### Meta-analysis of prevalence

Of the included studies, 25 were included in the meta-analysis of prevalence (25, 32–55). A sensitivity analysis was conducted to evaluate the impact of including the study conducted by Boivin et al.(56), which reported a very high prevalence of 98%. This study was excluded from the meta-analyses as it altered the size and precision of the results, as it focused only on patients who were hospitalised and prescribed lithium. The outcomes of the sensitivity analysis are included in the Supplementary Material.

Table 2 shows the pooled prevalence of CKD among people with SMI. Subgroup analyses were conducted to examine how prevalence differed across CKD stage, SMI diagnoses, lithium exposure, setting, and year of publication. The overall pooled prevalence of CKD among people with SMI was 8% (95% CI = 4%, 12%). Studies which collected data only from people with schizophrenia had the lowest prevalence of CKD (2%, 95% CI = 1%, 5%), compared to studies where data were collected only from people with bipolar disorder (15%, 95% CI = 6%, 26%). Additionally, studies reporting on the prevalence of CKD only among people with SMI exposed to lithium had a substantially higher prevalence (29%, 95% CI = 17%, 44%) compared to studies reporting mixed exposure cohorts (7%, 95% CI = 3%, 11%). Another sensitivity analysis was conducted to determine whether the exclusion of studies which used albuminuria or self-report to identify CKD influenced the results, but this had little effect (7%, 95% CI = 4%, 11%).

**Figure 2:**
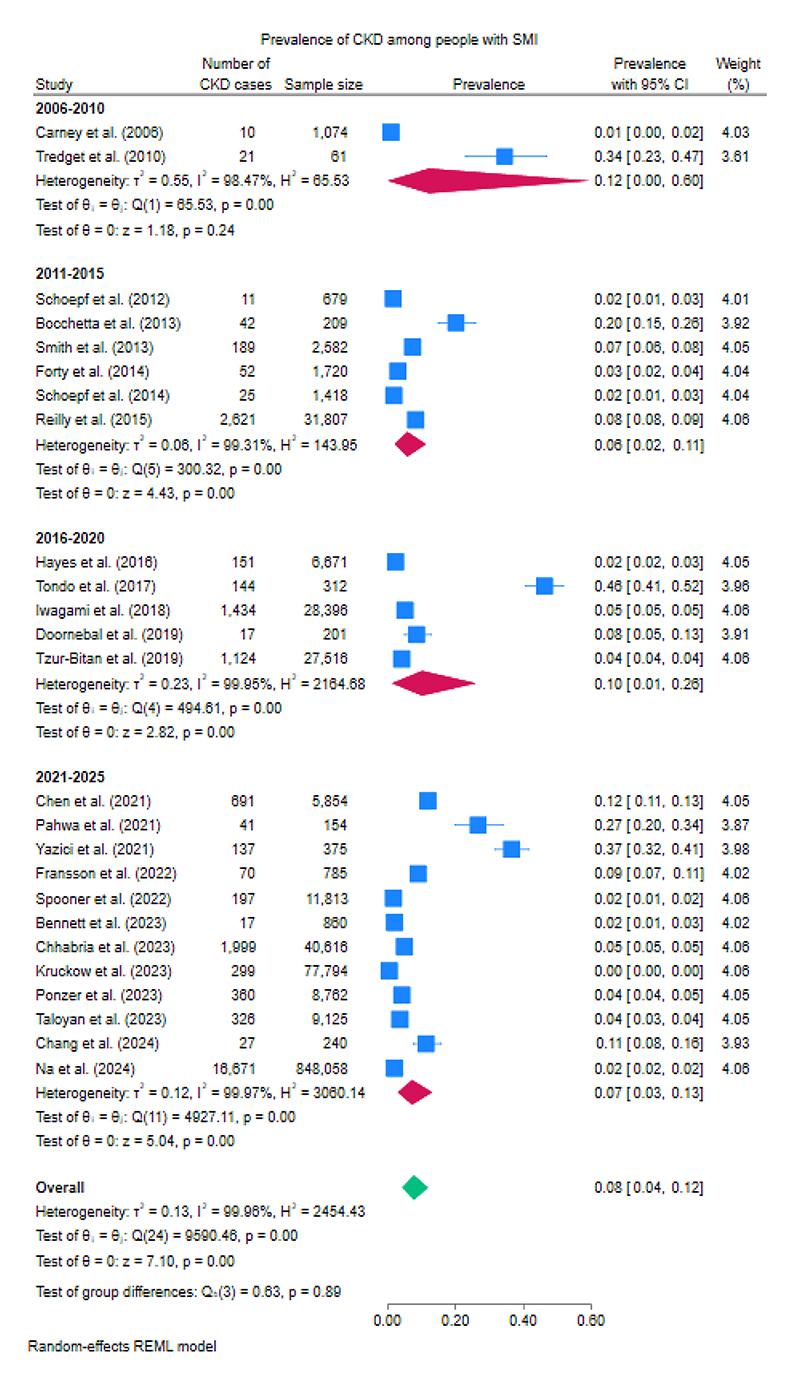
Pooled prevalence of CKD among people with SMI 166x293mm (72 x 72 DPI)

The forest plot for the meta-analysis of prevalence, divided by year of publication, is shown in Figure 2.

### Meta-analysis of incidence risk

Thirteen studies were included in the meta-analysis of incidence risk (39, 57–68). A sensitivity analysis was conducted to determine whether the exclusion of studies which used biopsy or self-report to identify CKD influenced the results. However, this did not result in a substantial change in the estimate (4%, 95% CI= 0%, 11%).

Table 3 reports the pooled incidence risk of CKD among people with SMI. Subgroup analyses were conducted to examine how incidence differed across length of follow-up, CKD stage, SMI diagnoses, lithium exposure, setting, and year of publication. The overall pooled incidence risk of CKD among people with SMI was 5% (95% CI= 3%, 7%). Studies which reported the incidence of CKD stage 3 and above had a higher incidence risk (7%, 95% CI = 2%, 13%) compared to studies which reported the incidence of CKD and did not specify a stage (3%, 95% CI = 1%, 6%). Incidence risk was highest at 16-20 years (8%, 95% CI = 5%, 12%) and 26-30 years follow-up (7%, 95% CI = 0%, 26%), although most studies had fewer than 20 years of follow-up.

**Figure 3:**
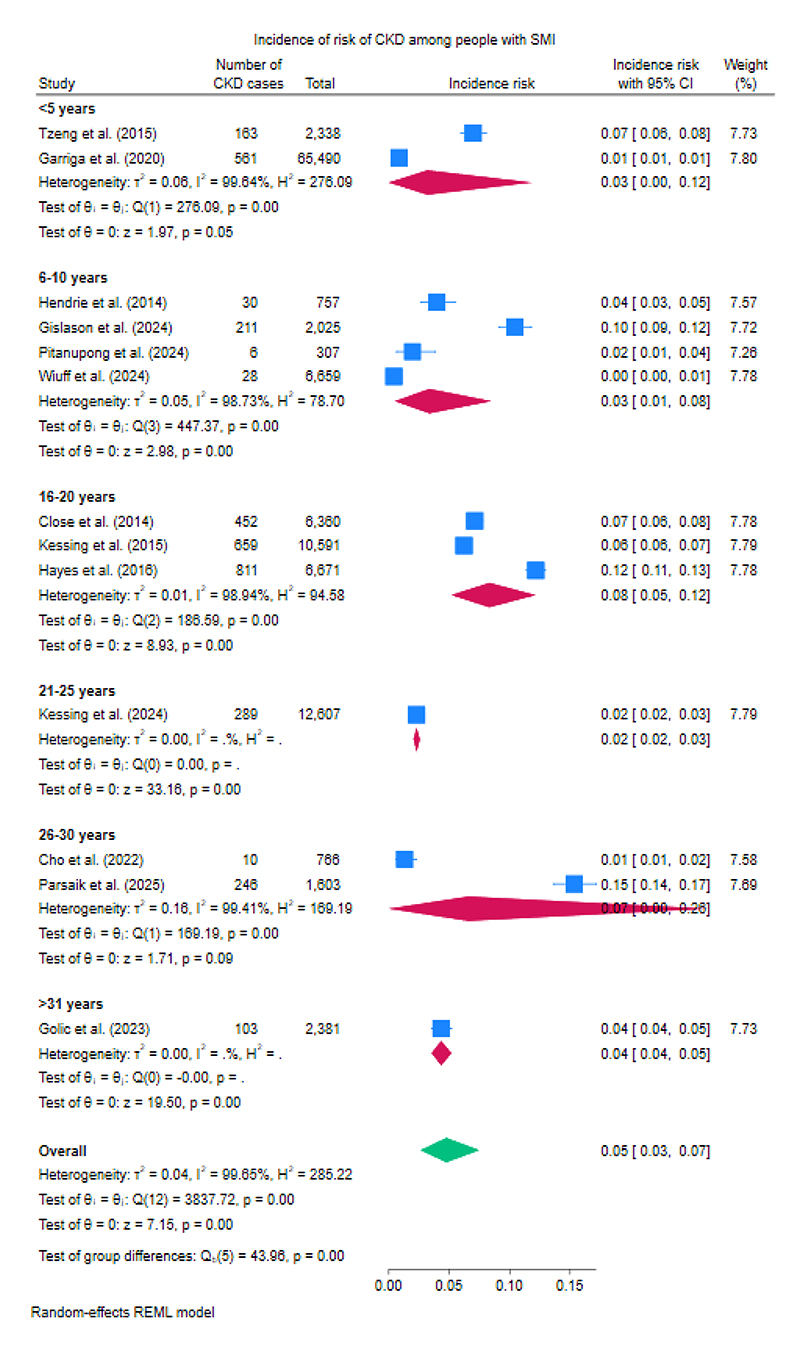
Pooled incidence risk of CKD among people with SMI 171x293mm (72 x 72 DPI)

Figure 3 shows the forest plot for the meta-analyses of incidence risk, according to the length of follow-up.

### Meta-analysis of incidence rates

**Figure 4:**
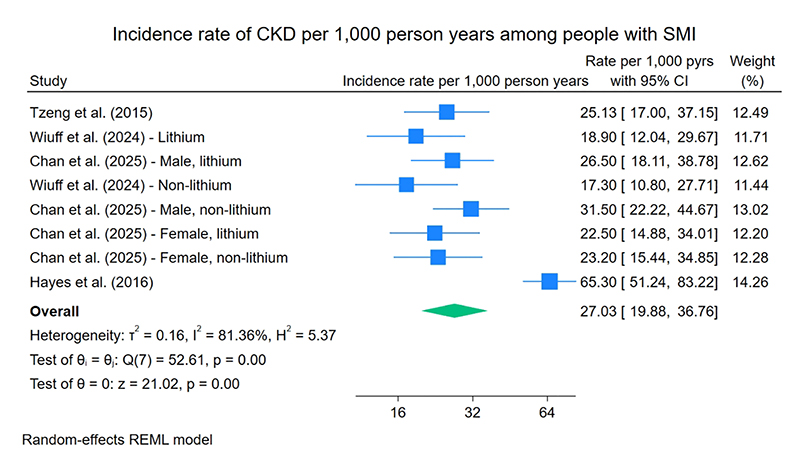
Pooled incidence rates (per 1,000 person-years) of CKD among people with SMI 524x293mm (72 x 72 DPI)

Four studies were pooled in a meta-analysis of incidence rates (39, 61, 67, 69), with one study reporting rates specifically in people exposed to lithium and not exposed to lithium (67), and another reporting incidence rates specific to lithium exposure and sex (69). Therefore, eight different rates were pooled across four studies. The pooled incidence rate was 26.83 cases (95% CI, 18.66-38.58) per 1,000 person-years. Figure 4 shows the forest-plot for the meta-analyses of incidence rates.

### Meta-analysis of odds ratios

Eleven studies were included in the meta-analysis of odds ratios (OR) for the prevalence of CKD among people with SMI (32, 36–38, 41, 43, 47, 48, 53, 55, 70). Table 4 reports the pooled OR of CKD among people with SMI compared to people without SMI or the general population. Subgroup analyses were conducted to examine how odds differed across CKD stage, SMI diagnoses, and year of publication. Only one study reported the odds of CKD among people with SMI and lithium exposure compared to a population without SMI (41), and only one study reported the odds of CKD in an inpatient population with SMI compared to people without SMI(38), therefore a subgroup analysis of odds ratios could not be conducted across lithium exposure and setting. A sensitivity analysis was performed to see whether removing self-report of CKD diagnosis influenced the pooled odds ratio (37), but this had minimal effect (pooled OR = 2.25, 95% CI = 1.62, 3.11).

The overall pooled odds ratio showed significantly higher odds of CKD among people with SMI than those without SMI (2.34, 95% CI = 1.68, 3.25). These increased odds were observed across all subgroup analyses.

### Meta-analysis of hazard ratios

Table 5 reports the pooled hazard ratios of CKD among people with SMI (52, 59, 61, 62, 67). Due to the limited number of studies which provided HR data (n=5), only one subgroup analysis was conducted to explore the overall pooled HR for CKD among people with SMI. The pooled HR was 1.96 (95% CI = 1.13, 3.40).

**Figure 6:**
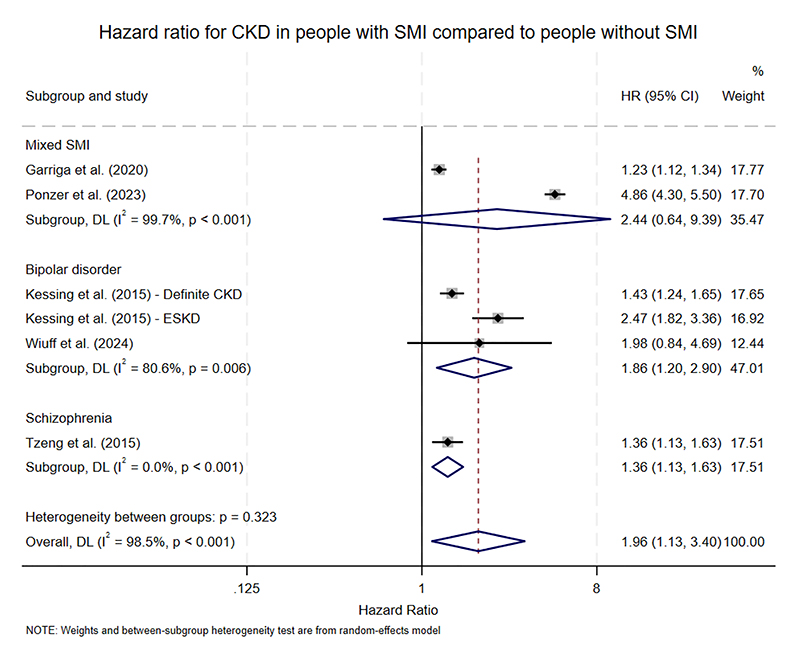
Pooled Hazard Ratio of CKD among people with SMI compared to the general population 403x293mm (72 x 72 DPI)

Figure 6 shows the forest plot for the meta-analyses of the hazard ratios, according to SMI diagnosis.

### Studies excluded from the meta-analyses

In total, eight studies were included in the review but excluded from the meta-analyses (71–78). The primary reason for exclusion was clinical heterogeneity (n = 5). Of these five (69, 72, 73, 77, 78), three studies reported the incidence of diabetic kidney disease among people with SMI and type 2 diabetes. Chan et al. (2021) (75) found an incidence risk of 6.1% for diabetic kidney disease among people with schizophrenia, which was lower among people with diabetes who did not have schizophrenia (adj OR = 0.91, 95% CI = 0.82 – 1.01). In contrast, Scheuer et al. (2022) (77) found that people with SMI (either bipolar disorder or schizophrenia) had an incidence rate of 30.55 per 1000 person-years for diabetic kidney disease. The incidence rate ratio was significantly higher compared to people without severe mental illness (IRR = 1.15, 95% CI = 1.12-1.18), even after adjusting for substance use and co-morbidities (IRR = 1.08, 95% CI = 1.05-1.12).

The remaining three studies were excluded from the analysis because of methodological heterogeneity, this included only reporting incidence of kidney failure among a cohort of people with SMI who already had CKD (71), reported ratio comparisons that could not be pooled (74), and used hospitalisation episodes as the denominator (as opposed to the population sample size or person-years at risk) (76).

### Quality of evidence - GRADE

We judged the overall quality of evidence available to be low. The risk of bias in individual studies was assessed as low, as most included studies were of high or moderate quality. However, our judgement was downgraded due to the indirectness of the evidence (with over half of the studies not having a primary aim to determine the prevalence or incidence of CKD in people with SMI), the high level of heterogeneity in the included studies, the imprecision across the confidence intervals of the included studies, and the lack of representation of prevalence estimates from important settings (such as from Low and middle income countries (LMICs)) and estimates according to specific factors such as ethnicity, gender and antipsychotic medication prescriptions.

## Discussion

This is the first systematic review to evaluate CKD prevalence, incidence and risk among people with SMI. We found that there was an 8% prevalence of CKD (all stages) in included studies, with the highest prevalence of CKD among populations who had a history of lithium exposure (29%) or a diagnosis of bipolar disorder (15%). The pooled incidence risk of CKD was 5%, and the pooled incidence rate was 26.8 per 1,000 person-years.

The pooled prevalence of CKD in this meta-analysis is lower than that in meta-analyses of prevalence among the general population, where the estimated prevalence of CKD is approximately 10.8% (79). In this review, the incidence risk is hard to compare as most studies only reported the incidence risk as a proportion without contextualising it within a specific follow-up time; however, the pooled incidence rate of 26.8 per 1,000 person-years is higher than findings from epidemiological studies conducted in the general population, for example, a study conducted in the Netherlands reported an incidence rate for CKD of approximately 12.13 per 1,000 person-years (80).

A further contrast to the lower pooled prevalence of CKD in comparison to the literature on the general population is that in studies where there were direct comparisons between the general populations and people with SMI, people with SMI consistently had higher odds of CKD. The pooled OR for CKD was 2.33, and while this was highest in studies focused on populations with bipolar disorder and schizoaffective disorder (OR = 2.78), studies which focused only on people with schizophrenia also had significantly higher odds of CKD (OR = 1.98, 95% CI = 1.56, 2.36). One reason for the comparatively low pooled prevalence estimate, but consistently higher odds and hazard of CKD among people with SMI compared to the general population, could be the method of identification of CKD. Most included studies relied on ICD diagnostic codes within population-level data (such as medical records and registries) to identify cases of CKD. While this approach allows the collection of data from a representative sample over long periods of follow-up, it has been shown to underestimate the prevalence or incidence of CKD(81, 82), especially in underserved and marginalised groups who do not have opportunistic testing. It is therefore recommended that a combination of clinical values, such as eGFR calculations and albuminuria based on medical records, be used in conjunction with diagnostic codes to ensure a more accurate estimate of the prevalence or incidence of CKD at a population level (81).

The prevalence of CKD differed according to the staging of CKD, which could also reflect the methods of identification required for staging. In studies where the stage was not reported (typically population-level research using diagnostic codes), the pooled prevalence was 4%, while the prevalence for CKD stage 3 and above was 12%. This was also reflected in the pooled incidence risk, where the pooled incidence risk for non-specified CKD was 3%, while for CKD stage 3 and above, the incidence risk was 7%. As CKD stage 3 and above has a lower prevalence and incidence than CKD of any stage in the general population (12), likely, this is due to better identification when staging CKD in epidemiological studies.

The higher odds of CKD among people with SMI compared to the general population likely result from a multitude of different factors. We found that the odds were highest among populations where mood stabilisers, such as lithium, would be indicated (for example, bipolar disorder and schizoaffective disorder). This is not surprising considering the established risk of lowered kidney function in long-term lithium treatment (17) and the associated risk of AKI (19). Additionally, increased screening in that population may lead to increased detection of CKD, particularly in the earlier stages, which is underdiagnosed in the general population (41). Importantly, the higher odds remained in populations where patients may not be exposed to lithium, such as in Schizophrenia. One study conducted by Iwagami et al. (2018) (41) highlighted the relative increased prevalence of CKD among people with SMI compared to the general population remained even when patients with any history of lithium exposure were excluded. This provides evidence that other risk factors are contributing to this risk. Second-generation antipsychotics have been associated with an increased risk of CKD (83), while studies examining the relative risk of CKD across first and second-generation antipsychotics identified combination treatment as being associated with the highest risk (84). While there is a lack of research evaluating the contributing factors associated with CKD risk among people with SMI, some studies identified in this review suggest that higher rates of conditions like diabetes are not solely responsible for this increase in CKD risk, with, Scheuer et al. (2022) finding a significantly higher rate of diabetic nephropathy even after adjusting for substance use and multi-morbidities (77). Therefore, further research is needed to understand the factors driving this increased risk and to identify effective strategies for reducing it.

Despite this increased risk of CKD among people with SMI, there is evidence that people with SMI and CKD experience worse outcomes compared to people without SMI or those in the general population(16). This includes higher mortality risk among people with SMI and kidney failure (85), limited access to specialist kidney care (86), transplant assessment (87, 88), and kidney replacement therapies(43), and higher rates of emergency hospitalisation(89). There is also evidence to suggest that people with SMI and CKD experience worse outcomes than people with SMI who do not have CKD, including associations between CKD and suicide risk (90)(91).

### Strengths and Limitations

There are several strengths and limitations to this review, as well as to the overall evidence base. There was a relatively small number of articles included in the meta-analyses, particularly for meaningful subgroup analysis. Therefore, the confidence intervals in the meta-analyses lacked precision, although we were able to demonstrate an increased risk of CKD across studies which reported relevant ratios. There was a lack of research conducted in LMICs, and most studies did not provide a breakdown of CKD prevalence, incidence or risk, according to SMI diagnoses, antipsychotic medication, gender, age, BMI or multi-morbidities, limiting our ability to explore potential contributing factors to the risk of CKD. However, we were able to conduct subgroup analyses according to method of CKD identification, lithium exposure, and staging of CKD, highlighting some potential limitations in the evidence base that can be addressed in future research. We were able to conduct separate meta-analyses to report prevalence, incidence risk and incidence rate, in addition to odds and hazard ratios to provide a more comprehensive overview of the epidemiological literature on this relationship. Several pooled studies were conducted in the same country (for example, the UK), and the reliance on national datasets may result in overlapping samples that could have led to an inaccurate measure of precision for the pooled estimates.

This review was also focused specifically on studies which included people diagnosed with SMI. We did not include all studies which reported the risk of CKD among people receiving lithium treatment (if they did not report the diagnoses of included participants). Additionally, not all included studies consistently reported lithium exposure or provided a breakdown of CKD risk across lithium-exposure groups (although this was analysed when available). Therefore, the findings reported in this review may not accurately reflect the degree of risk associated with lithium exposure on its own. Finally, the studies did not account for the impact of screening in prevalence estimates of CKD, which is particularly important in studies focused on people receiving lithium treatment, where regular screening of kidney function is part of the care pathway (17). Despite these limitations, this is the first systematic review to provide a pooled prevalence estimate of CKD among people with SMI.

## Conclusion

While the prevalence estimates in this review are lower than in the general population, meta-analyses of incidence rates, odds and hazard ratios demonstrate that people with SMI are at a significantly higher risk of CKD compared to people without SMI. Lithium is known to play a role in the increased risk among people with bipolar and schizoaffective disorders. The evidence for the relationship between CKD and SMI in general is limited and imprecise, and while psychiatric medication and high rates of type 2 diabetes may contribute to the risk, the drivers of this inequality are under-researched. Further research is needed to identify any potentially modifiable risk factors and establish the global prevalence and incidence of CKD in people with SMI.

## Supplementary Material

Supplementary Material

## Figures and Tables

**Figure 5:**
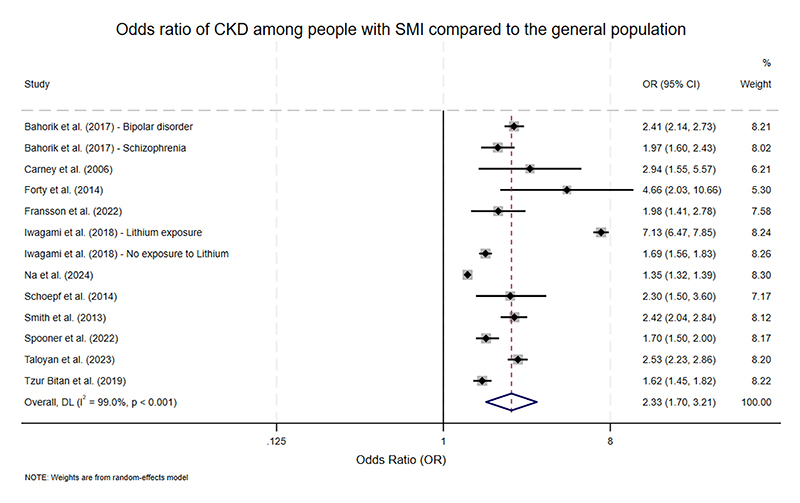
Pooled OR of CKD among people with SMI compared to the general population 403x293mm (72 x 72 DPI)

**Table 1:** Overview of included studies.

Author	Sample size *	SMI	Setting	Country	WB Income **	Study design	CKD stage reported ***	Comparison with the general population	CKD outcomes reported	JBI Quality appraisal	Pooled in a meta-analysis
Presne et al. 2003 (71)	74	Bipolar disorder and schizoaffective disorder	Community	France	HIC	Cohort	Stage 5 (ESRD)	No	Incidence risk	Low	No - reported kidney failure among participants already with CKD.
Carney et al. 2006 (32)	1074	Schizophrenia	Population level	USA	HIC	Cross sectional	CKD stage 3>	Yes	Prevalence and odds ratio	High	Yes
Tredget, Kirov and Kirov, 2010 (33)	61	Bipolar disorder	Community	UK	HIC	Cross sectional	CKD stage 3>	No	Prevalence	High	Yes
Schoepf et al 2012 (34)	679	Schizophrenia	Inpatient	UK	HIC	Cohort	NR	Yes	Prevalence and risk ratio	High	Yes
Bocchetta et al. 2013 (35)	209	Bipolar disorder	NR	Italy	HIC	Cross sectional	CKD stage 3>	No	Prevalence	High	Yes
Smith et al. 2013 (36)	2582	Bipolar disorder	Population level	UK	HIC	Cross sectional	NR	Yes	Prevalence and odds ratio	High	Yes
Close et al. 2014 (57)	6360	Bipolar disorder	Population level	UK	HIC	Cohort	CKD stage 3>	No	Incidence risk	High	Yes
Forty et al. 2014 (37)	785	Bipolar disorder and schizoaffective disorder	NR	UK	HIC	Cross sectional	NR	Yes	Prevalence and odds ratio	Low	Yes
Hendrie et al., 2014 (58)	31588	Schizophrenia	Community	USA	HIC	Cohort	NR	Yes	Incidence risk	High	Yes
Schoepf et al 2014 (38)	1418	Schizophrenia	Inpatient	UK	HIC	Cohort	NR	Yes	Prevalence and odds ratio	High	Yes
Kessing et al, 2015 (59)	10591	Bipolar disorder	Population	Denmark	HIC	Cohort	NR	Yes	Incidence risk and hazard ratio	High	Yes
Reilly et al 2015 (25)	31807	Schizophrenia, bipolar disorder and unspecified psychosis	Population	UK	HIC	Cohort	NR	Yes	Prevalence	Moderate	Yes (most recent annual prevalence data)
Tzeng et al. 2015 (61)	2338	Schizophrenia	Population	Taiwan	HIC	Cohort	NR	Yes	Incidence risk, incidence rate and hazard ratio	Moderate	Yes
Hayes et al. 2016 (39)	6671	Bipolar disorder	Population	UK	HIC	Cohort	CKD stage 3>	No	Prevalence and Incidence rate	Moderate	Yes
Bahorik et al. 2017 (70)	25090	Bipolar disorder and schizophrenia	Population	USA	HIC	Cross sectional	NR	Yes	NR (Reports Odds ratio only)	High	Yes
Tondo et al 2017 (40)	312	Bipolar disorder	NR	International	HIC and UMIC	Cohort	CKD Stage 3-4	No	Prevalence	Low	Yes (prevalence of stage 3-4 at final follow-up point)
Iwagami et al, 2018 (41)	28396	Bipolar disorder, schizophrenia and non-organic psychosis	Population	UK	HIC	Cross sectional	NR	Yes	Prevalence and odds ratio	High	Yes
Doornebal et al. 2019 (42)	201	Bipolar disorder and schizoaffective disorder	Community	The Netherlands	HIC	Cross sectional	CKD stage 3>	No	Prevalence	Moderate	Yes
Tzur-Bitan et al., 2019 (43)	27516	Schizophrenia	Population	Israel	HIC	Cross sectional	NR	Yes	Prevalence and odds ratio	High	Yes
Attar et al. 2020 (72)	1008	Schizophrenia	Population	Sweden	HIC	Cohort	CKD Stage 1-3	Yes	Prevalence	High	No (only reports prevalence among people with SMI who have had a myocardial infarction)
Garriga et al. 2020 (62)	65490	Psychosis, schizophrenia and bipolar disorder	Population	UK	HIC	Cohort	NR	Yes	Incidence risk and hazard ratio	High	Yes
Toender et al 2020 (73)	3529	Schizophrenia	Population	Denmark	HIC	Cohort	NR	Yes	Incidence risk	High	No (only reports incidence among people with SMI and diabetes)
Uju et al. 2020 (74)	144	Schizophrenia	Inpatient	Japan	HIC	Cross-sectional	NR	No	NR (Prevalence risk ratio reported)	Low	No (Prevalence risk ratio compared between psychosis and affective disorders group)
Chan et al, 2021 (75)	7001	Schizophrenia	Population	Hong Kong	HIC	Cohort	NR	Yes	Incidence risk and odds ratio	High	No (only reports the incidence risk of diabetic nephropathy)
Chen et al. 2021 (44)	5854	Bipolar disorder	Population	Taiwan	HIC	Cohort	NR	No	Prevalence	High	Yes
Pahwa et al 2021 (45)	154	Bipolar disorder	Community and inpatient	USA	HIC	Cohort	CKD Stage 3>	No	Incidence risk	High	Yes
Yazici et al 2021 (46)	375	Bipolar disorder and schizoaffective disorder	Community	Turkey	UMIC	Cross sectional	CKD Stage 1-3	No	Prevalence	Moderate	Yes
Cho et al. 2022 (63)	766	Bipolar disorder, schizophrenia and psychotic disorders	NR	South Korea	HIC	Cohort	CKD Stage 3>	No	Incidence risk	High	Yes
Fransson et al. 2022 (47)	785	Bipolar disorder and schizoaffective disorder	Population	Sweden	HIC	Cross sectional	CKD Stage 3>	Yes	Prevalence and odds ratio	High	Yes
Henriques et al, 2022 (76)	20807	Bipolar disorder	Population	Portugal	HIC	Cohort	NR	No	Prevalence	Moderate	No (Denominator of the study was hospitalisation episodes, not individual patients)
Scheuer et al. 2022 (77)	30102	Schizophrenia and bipolar disorder	Population	Denmark	HIC	Cohort	NR	Yes	Incidence Rate and Incidence Risk Ratio	High	No (only reporting incidence rate of diabetic nephropathy)
Spooner et al. 2022 (48)	11813	Schizophrenia and bipolar disorder	Population	Australia	HIC	Cross sectional	NR	Yes	Prevalence and odds ratio	Moderate	Yes
Bennett et al. 2023 (49)	860	Bipolar disorder, schizophrenia, major depression with psychosis, other psychotic disorders	Community	USA	HIC	Cross sectional	NR	No	Prevalence	High	Yes
Boivin e al. 2023 (56)	248	Bipolar disorder and schizophrenia	Inpatient	France	HIC	Cohort	CKD Stages 1-5	No	Prevalence	Low	Yes
Chhabria et al 2023 (50)	40616	Bipolar disorder	Population	USA	HIC	Cross sectional	NR	No	Prevalence	High	Yes
Golic et al. 2023 (64)	2381	Bipolar disorder	Population	Sweden	HIC	Cohort and case control	CKD Stage 4>	Yes	Incidence	High	No (Only reports SMI diagnoses for cases of CKD, unclear breakdown of cohort)
Kruckow et al. 2023 (51)	77794	Schizophrenia	Population	Denmark	HIC	Cohort	NR	No	Prevalence	High	Yes
Ponzer et al. 2023 (52)	8762	Bipolar disorder	Population	Finland	HIC	Cohort	NR	Yes	Prevalence and hazard ratio	Low	Yes
Taloyan et al 2023 (53)	9 125	Schizophrenia and bipolar disorder	Population	Sweden	HIC	Cross sectional	NR	Yes	Prevalence and odds ratio	High	Yes
Gislason et al., 2024 (65)	3198	Schizophrenia, bipolar disorder and schizoaffective disorder	Population	Iceland	HIC	Cohort	CKD Stage 3>	No	Incidence risk	High	Yes
Kessing et al, 2024 (60)	12, 607	Bipolar disorder	Population	Denmark	HIC	Cohort	NR	No	Incidence risk	High	Yes
Na et al. 2024 (55)	848058	Schizophrenia, bipolar disorder and depression with psychosis	Population	South Korea	HIC	Cross sectional	NR	Yes	Prevalence and odds ratio	High	Yes
Pitanupong et al 2024 (66)	307	Bipolar disorder and schizoaffective disorder	Community	Thailand	UMIC	Cohort	CKD Stage 3>	No	Incidence risk and rate	Moderate	Yes
Wiuff et al 2024 (67)	1646	Bipolar disorder	Population	Denmark	HIC	Cohort	NR	Yes	Incidence risk, incidence rate and hazard ratio	High	Yes
Chan et al 2025 (69)	7029	Bipolar disorder	Population	Hong Kong	HIC	Cohort	CKD Stage 3>	No	Incidence rate	High	Yes
Chang et al, 2025 (54)	240	Schizophrenia	NR	Taiwan	HIC	Cross-sectional	CKD Stages 1-5	No	Prevalence	Low	Yes
Fransson et al 2025 (78)	168	Bipolar disorder and schizoaffective disorder	Population	Sweden	HIC	Cohort	CKD Stages 1-5	No	Prevalence	High	No (only reports data from patients who discontinued lithium)
Parsaik et al 2025 (68)	1603	Bipolar disorder	Population	USA	HIC	Cohort	CKD Stage 3>	No	Incidence risk	High	Yes

* Denominator sample size for reporting prevalence or incidence (excluding controls used in any comparisons)

**Table 2:** Pooled prevalence of CKD among people with SMI

Variable	Number of studies Pooled	CKD prevalence (95% CI)	I^2^ statistic
**Overall pooled prevalence** *	25	8% (4%, 12%)	99.96%
**By CKD stage/ definition**			
CKD - Stage not reported	15	4% (2%, 5%)	99.85%
CKD Stage 1-3	1	37% (32%, 41%)	-
CKD Stage 1-5	1	11% (8%, 16%)	-
CKD Stage >3	7	12% (4%, 23%)	99.03%
CKD Stage 3-4	1	46% (41%, 52%)	-
**By SMI**			
Bipolar disorder	9	15% (6%, 26%)	99.87%
Schizophrenia Bipolar disorder and schizoaffective	6	2% (1%, 5%)	99.70%
disorder	4	12% (2%, 28%)	99.03%
Mixed SMI	6	3% (2%, 6%)	99.80%
**By lithium exposure**			
Lithium exposed	5	29% (17%, 44%)	95.89%
Mixed exposure	8	7% (3%, 11%)	99.44%
Not reported	12	3% (2%, 5%)	99.89%
**By setting**			
Population	14	4% (3%, 6%)	99.89%
Community	4	17% (3%, 39%)	98.64%
Inpatient	2	2% (1%, 2%)	0.01%
Inpatient and community	1	27% (20%, 34%)	-
Not reported	4	7% (2%, 27%)	98.94%
**By the method of CKD identification**			
Diagnostic code	16	4% [2%, 6%]	99.92%
eGFR calculation	7	17% [8%, 29%]	98.82%
eGFR calculation and albuminuria	1	37% [32%, 41%]	-
Self-report	1	3% [2%, 4%]	-
**By year of publication**			
2006-2010	2	12% (0%, 60%)	98.47%
2011-2015	6	6% (2%, 11%)	99.31%
2016-2020	5	10% (1%, 26%)	99.95%
2021-2025	12	7% (3%, 13%)	99.97%

**Table 3:** The pooled incidence risk of CKD among people with SMI.

Variable	Number of studies	CKD incidence risk (95% CI)	I^2^ statistics
**Overall pooled incidence risk**	13	5% (3%, 7%)	99.65%
**By length of follow-up**			
<5 years	2	3% (0%, 12%)	99.64%
6-10 years	4	3% (1%, 8%)	98.73%
16-20 years	3	8% (5%, 12%)	98.94%
21-25 years	1	2% (2%, 33%)	-
26-30 years	2	7% (0%, 26%)	99.41%
>31 years	1	4% (4%, 5%)	-
**By CKD stage/ definition**			
CKD - Stage not reported	6	3% (1%, 6%)	99.65%
CKD Stage >3	6	7% (2%, 13%)	99.24%
CKD Stafe >4	1	4% (4%, 5%)	-
**By SMI**			
Bipolar disorder	7	6% (2%, 10%)	99.70%
Schizophrenia	2	5% (3%, 9%)	89.95%
Bipolar disorder and schizoaffective	1	2% (1%, 4%)	-
disorder Mixed SMI	3	3% (0%, 10%)	99.45%
**By lithium (n=8)**			
Lithium exposed only	5	5% (1%, 12%)	99.37%
Mixed exposure	4	5% (1%, 10%)	99.68%
Not reported	4	4% (2%, 8%)	99.37%
**By setting (n=8)**			
Population	10	6% (3%, 9%)	99.76%
Community	2	3% (1%, 5%)	63.91%
Not reported	1	1% (1%, 2%)	-
**By year of publication (n=8)**			
2011-2015	4	6% (5%, 7%)	90.60%
2016-2020	2	5% (0%, 22%)	99.94%
2021-2025	7	4% (1%, 8%)	99.44%

**Table 4:** Pooled odds ratios of CKD among people with SMI compared to people without SMI

Variable	Number of studies	Pooled OR (95% CI)	I^2^ statistic
**Overall pooled Odds Ratio (OR)**	11	2.33 (1.70, 3.21)	99.00%
**By CKD stage/ definition**			
CKD - Stage not reported	9	2.33 (1.63, 3.34)	99.20%
CKD Stage >3	2	2.19 (1.56, 3.07)	12.70%
**By SMI** *			
Bipolar disorder	2	2.41 (2.19, 2.66)	0.0%
Schizophrenia	4	1.92 (1.56, 2.36)	57.30%
Bipolar disorder and schizoaffective disoSysrder	2	2.78 (1.22, 6.33)	71.50%
Mixed SMI	4	2.34 (1.30, 4.21)	99.60%
**By year**			
2006-2010	1	2.94 (1.55, 5.57)	-
2011-2015	3	2.50 (2.02, 3.09)	17.00%
2016-2020	3	2.48 (1.34, 4.57)	99.30%
2021-2025	4	1.83 (1.29, 2.59)	97.20%

* Bahorik et al.(2017) reported odds ratios separately for bipolar disorder and schizophrenia, while Iwagami et al. (2018) reported odds ratios separately for those exposed to lithium and those who had not been exposed to lithium.

**Table 5:** Pooled HR of CKD among people with SMI compared to the general population

Variable	Number of studies	Pooled HR (95% CI)	I^2^statistic
**Overall pooled Hazard Ratio**	5	1.96 (1.13, 3.40)	98.50%
By SMI*			
Bipolar disorder	2	1.86 (1.20, 2.90)	80.60%
Schizophrenia	1	1.36 (1.13, 3.40)	-
Mixed SMI	2	2.44 (0.64, 9.39)	99.70%

## Data Availability

Data availability is not applicable as no new data were created or analysed in this study.
