# Supplementary material for: Prevalence of chronic kidney disease in people with severe mental illness: A systematic review and meta-analysis
Source: Br J Psychiatry. Author manuscript; Available in PMC 2026 Jun 15. (PMC7619184; doi:10.1192/bjp.2026.10659)

Supplementary Material

Supplementary Material 1 – Search Strategies

Medline

Chronic Kidney Disease (CKD) search string (Combined with OR)

1. exp Renal Dialysis/
2. h?emodialysis.tw,kf.
3. h?emofiltration.tw,kf.
4. h?emodiafiltration.tw,kf.
5. dialysis.tw,kf.
6. (Peritoneal adj1 (dialysis)).tw,kf.
7. (PD or CAPD or CCPD or APD).tw,kf.
8. Exp Renal Insufficiency/
9. Exp Kidney Failure/
10. exp Renal Insufficiency, Chronic/
11. Exp Kidney Diseases/
12. Uremia/
13. (endstage adj1 (renal or kidney)).tw,kf
14. (ESRF or ESKF or ESRD or ESKD).tw,kf
15. (chronic adj1 (kidney or renal)).tw,kf
16. (CKF or CKD or CRF or CRD).tw,kf.
17. (predialysis).tw,kf.
18. (renal adj1 (transplant* or graft)).tw,kf.
19. (kidney adj1 (transplant* or graft)).tw,kf.

Severe mental illness (SMI) search string (Combined with OR)

1. exp Schizophrenia/
2. Exp Affective disorders, psychotic/
3. Exp Bipolar disorder/
4. (bipolar adj1 (disorder* or disease* or illness*)).tw,kf.
5. paranoid disorders/
6. exp psychotic disorders/
7. schizo*.tw,kf.
8. (mani* adj3 depress*).tw,kf.
9. (psychotic* adj3 depress*).tw,kf.
10. (severe* adj3 affective*).tw,kf.
11. (severe* adj3 mental*).tw,kf.
12. (severe* adj3 depress*).tw,kf.
13. (psychos#s adj3 depress*).tw,kf.
14. (serious* adj3 affective*).tw,kf.
15. "serious mood*".tw,kf.
16. (serious* adj3 mental*).tw,kf.
17. (serious* adj3 depress*).tw,kf.

18. “Severe mental*”.tw,kf.
19. Severe adj1 (mental* or depress*).tw,kf.
20. “Schizoaffective disorder”.tw,kf
21. exp Antipsychotic Agents/
22. exp Antimanic Agents/
23. exp Psychotropic Drugs/

Study design search string (Combined with OR)

1. exp epidemiologic studies/
2. exp epidemiology/
3. epidemiolog*.tw,kf.
4. exp prevalence/
5. prevalence.tw,kf.
6. exp incidence/
7. incidence.tw,kf.
8. Exp Observational Study/
9. observational.tw,kf.
10. Longitudinal Studies/
11. longitudinal.tw,kf.
12. Case-Control Studies/
13. Exp Cross-Sectional Studies/
14. Exp Cohort Studies/
15. “cohort”.tw,kf.
16. Risk/ or Risk Factors/

CKD AND SMI AND Study design combined with AND.

PsycInfo

Chronic Kidney Disease (CKD) search string (Combined with OR)

1. exp Dialysis/
2. h?emodialysis.tw.
3. h?emofiltration.tw.
4. h?emodiafiltration.tw.
5. dialysis.tw.
6. (Peritoneal adj1 dialysis).tw.
7. exp Kidney Diseases/
8. exp Hemodialysis/
9. (endstage adj1 (renal or kidney)).tw.
10. (ESRF or ESKF or ESRD or ESKD).tw.
11. (chronic adj1 (kidney or renal)).tw.
12. (CKF or CKD or CRF or CRD).tw.
13. predialysis.tw.
14. (renal adj1 (transplant* or graft)).tw.
15. (kidney adj1 (transplant* or graft)).tw.

Severe mental illness (SMI) search string (Combined with OR)

16. exp "Schizophrenia (Disorganized Type)"/ or exp Schizophrenia/ or exp Acute Schizophrenia/ or exp Paranoid Schizophrenia/ or exp Undifferentiated Schizophrenia/ or exp Catatonic Schizophrenia/
17. exp Bipolar Disorder/ or exp Psychosis/ or exp Major Depression/ or exp Affective Psychosis/
18. (bipolar adj1 (disorder* or disease* or illness*)).tw.
19. exp Paranoid Psychosis/
20. schizo*.tw.
21. (mani* adj3 depress*).tw.
22. (psychotic* adj3 depress*).tw.
23. (severe* adj3 affective*).tw.
24. (severe* adj3 mental*).tw.
25. (severe* adj3 depress*).tw.
26. (psychos#s adj3 depress*).tw.
27. (serious* adj3 affective*).tw.
28. "serious mood*".tw.
29. (serious* adj3 mental*).tw.
30. (serious* adj3 depress*).tw.
31. "Severe mental*".tw.
32. (Severe adj1 (mental* or depress*)).tw.
33. "Schizoaffective disorder".tw.
34. exp Neuroleptic Drugs/
35. exp Mood Stabilizers/

Study design search string (Combined with OR)

36. exp Epidemiology/
37. epidemiolog*.tw.
38. prevalence.tw.
39. incidence.tw.
40. observational.tw.
41. longitudinal.tw.
42. exp Longitudinal Studies/
43. "case-control".tw.
44. "cross-sectional".tw.
45. cohort.tw.
46. exp Risk Factors/

CKD, SMI and study design search strings combined with AND

EMBASE

Chronic Kidney Disease (CKD) search string (Combined with OR)

1. 1 or 2 or 3 or 4 or 5 or 6 or 7 or 8 or 9 or 10 or 11 or 12 or 13 or 14 or 15 or 16 or 17 or 18 or 19
2. (kidney adj1 (transplant* or graft)).tw,kf.
3. (kidney adj1 (transplant* or graft)).tw,kf.
4. (renal adj1 (transplant* or graft)).tw,kf.
5. predialysis.tw,kf.
6. (CKF or CKD or CRF or CRD).tw,kf.
7. (chronic adj1 (kidney or renal)).tw,kf.
8. (ESRF or ESKF or ESRD or ESKD).tw,kf.
9. (endstage adj1 (renal or kidney)).tw,kf.
10. exp uremia/
11. exp chronic kidney failure/
12. exp kidney failure/
13. exp kidney disease/
14. (Peritoneal adj1 dialysis).tw,kf.
15. dialysis.tw,kf.
16. h?emodiafiltration.tw,kf.
17. h?emofiltration.tw,kf.
18. h?emodialysis.tw,kf.
19. hemodialysis/ or hemodialysis patient/
20. exp dialysis/ or exp home dialysis/ or exp continuous ambulatory peritoneal dialysis/ or exp peritoneal dialysis/

Severe mental illness (SMI) search string (Combined with OR)

1. 21 or 22 or 23 or 24 or 25 or 26 or 27 or 28 or 29 or 30 or 31 or 32 or 33 or 34 or 35 or 36 or 37 or 38 or 39 or 40
2. exp mood stabilizer/ or exp lithium/
3. exp neuroleptic agent/
4. "Schizoaffective disorder".tw,kf.
5. (Severe adj1 (mental* or depress*)).tw,kf.
6. "Severe mental*".tw,kf.
7. (serious* adj3 depress*).tw,kf.
8. (serious* adj3 mental*).tw,kf.
9. "serious mood*".tw,kf.
10. (serious* adj3 affective*).tw,kf.
11. (psychos#s adj3 depress*).tw,kf.
12. (severe* adj3 depress*).tw,kf.
13. (severe* adj3 mental*).tw,kf.
14. (severe* adj3 affective*).tw,kf.
15. (psychotic* adj3 depress*).tw,kf.
16. (mani* adj3 depress*).tw,kf.
17. schizo*.tw,kf.
18. (bipolar adj1 (disorder* or disease* or illness*)).tw,kf.
19. exp psychosis/ or exp puerperal psychosis/ or exp acute psychosis/ or exp depressive psychosis/ or exp drug induced psychosis/ or exp treatment-resistant psychosis/ or exp schizoaffective psychosis/ or exp manic psychosis/ or exp affective psychosis/ or exp paranoid psychosis/ or exp endogenous psychosis/
20. exp bipolar disorder/

21. exp catatonic schizophrenia/ or exp schizophrenia spectrum disorder/ or exp latent schizophrenia/ or exp schizophrenia/ or exp residual schizophrenia/ or exp treatment-resistant schizophrenia/ or exp simple schizophrenia/ or exp paranoid schizophrenia/

Study design search string (combined with OR)

1. 42 or 43 or 44 or 45 or 46 or 47 or 48 or 49 or 50 or 51 or 52 or 53 or 54 or 55 or 56 or 57
2. exp cohort analysis/
3. "cohort".tw,kf.
4. exp cross-sectional study/
5. "cross-sectional".tw,kf.
6. exp case control study/
7. "case-control".tw,kf.
8. exp longitudinal study/
9. longitudinal.tw,kf.
10. exp observational study/ or exp observational method/
11. observational.tw,kf.
12. incidence.tw,kf.
13. exp standardized incidence ratio/ or exp cumulative incidence/ or exp incidence/
14. prevalence.tw,kf.
15. exp point prevalence/ or exp period prevalence/ or exp prevalence/
16. epidemiolog*.tw.
17. exp epidemiology/

CKD, SMI and study design search strings combined with AND

CINAHL

Chronic Kidney Disease (CKD) search string (Combined with OR)

1. (MH "Dialysis+") OR (MH "Dialysis Patients") OR (MH "Dialysis Centers") OR (MH "Peritoneal Dialysis+") OR (MH "Peritoneal Dialysis, Continuous Ambulatory") OR (MH "Home Dialysis") OR (MH "Hemodialysis+")
2. TX h?emodialysis
3. TX h?emofiltration OR TX h?emodiafiltration OR TX dialysis
4. TX Peritoneal dialysis
5. (MH "Polycystic Kidney, Autosomal Dominant") OR (MH "Kidney Diseases+") OR (MH "Kidney, Cystic+") OR (MH "Polycystic Kidney, Autosomal Recessive") OR (MH "Renal Osteodystrophy") OR (MH "Kidney Failure, Chronic+") OR (MH "Kidney Neoplasms+") OR (MH "Kidney Transplantation") OR (MH "Kidney Failure, Acute+")
6. TX (end stage renal disease)
7. TX (end stage kidney disease)
8. TX (chronic kidney disease) OR TX (chronic renal disease)
9. TX predialysis
10. TX (kidney transplant) OR TX (renal transplant) OR TX (kidney graft) OR TX (renal graft)

Severe mental illness (SMI) search string (Combined with OR)

12. (MH "Schizophrenia") OR (MH "Schizophrenia, Childhood") OR (MH "Schizophrenia, Treatment-Resistant") OR (MH "Schizoaffective Disorder") OR (MH "Risperidone") OR (MH "Olanzapine") OR (MH "Haloperidol") OR (MH "Catatonia")

13. (MH "Affective Disorders, Psychotic+") OR (MH "Psychotic Disorders+") OR (MH "Bipolar Disorder+")
14. TX schizophrenia OR TX (bipolar disorder) OR TX (schizoaffective disorder) OR TX psychosis
15. (MH "Paranoid Disorders")
16. TX schizo*
17. TX (mani* depression)
18. TX (psychotic depression) OR TX (severe affective) OR TX (severe mental illness) OR TX (serious mental illness)
19. TX psychos#s
20. (MH "Antipsychotic Agents+")
21. (MH "Lithium Carbonate") OR (MH "Cyclothymic Disorder") OR (MH "Antimanic Agents")

Study design search string (combined with OR)

23. (MH "Epidemiology+") OR (MH "Epidemiological Research+") OR (MH "Incidence")
24. TX epidemiolog*
25. TX prevalence OR TX incidence
26. TX (observational study) OR TX longitudional OR TX (case-control) OR TX cohort
27. (MH "Prospective Studies+") OR (MH "Concurrent Prospective Studies") OR (MH "Cross Sectional Studies") OR (MH "Case Control Studies+")
28. (MH "Risk Factors+") OR (MH "Relative Risk")

CKD, SMI and study design search strings combined with AND

Scopus

Chronic Kidney disease search string

dialysis OR h?emodialysis OR h?emofiltration OR h?emodiafiltration OR "peritoneal w/2 dialysis" OR {kidney failure} OR {renal failure} OR {end?stage kidney disease} OR {end?stage renal disease} {kidney disease} OR {chronic kidney disease} OR predialysis OR {renal w/2 transplant} OR {kidney w/2 transplant} OR {kidney w/2 graft}

AND

SMI search string

schizophrenia OR shizoaffective OR {schizoaffective disorder} OR {bipolar disorder} OR {psychotic depress*} OR psychos* OR {paranoid disorder*} OR {psychotic disorder*} OR {manic depress*} OR mania OR {severe w/2 affective*} OR {severe mental illness*} OR {serious mental illness*} OR {antimanic} OR {antipsychotic} OR {neuroleptic} OR {psychotropic} OR schizo*

AND

Study design search string

epidemiology OR epidemiolog* OR prevalence OR prevalen* OR incidence OR inciden* OR observational OR {observational study} OR longitudinal OR {longitudinal study} OR {case?control} OR {case?control study} OR cohort OR {cohort study} OR risk OR {risk factor*}

Web of science

dialysis OR h?emodialysis OR h?emofiltration OR h?emodiafiltration OR peritoneal OR "kidney failure" OR "renal failure" OR "end?stage kidney disease" OR "end?stage renal disease" OR "chronic kidney disease" OR "kidney disease" OR predialysis OR "renal transplant" OR "kidney transplant" OR "kidney graft" (All Fields) and schizophrenia OR schizoaffective OR schizo* OR "bipolar disorder" OR "psychotic depress*" OR psychos* OR "paranoid disorder*" OR "psychotic disorder*" OR "manic depress*" OR mania OR "severe affective*" OR "severe mental illness*" OR "serious mental illness*" OR antimanic OR antipsychotic OR neuroleptic OR psychotropic (All Fields) and epidemiology OR epidemiolog* OR prevalence OR prevalen* OR incidence OR inciden* OR observational OR "observational study" OR longitudinal OR "longitudinal study" OR "case?control" OR cohort OR risk OR "risk factor*" (All Fields)

Supplementary Material 2 – Sensitivity Analysis including Boivin et al. (2023)

Table S1. Pooled prevalence of CKD among people with SMI including Boivin et al. 2023

Variable	Number of studies Pooled	CKD prevalence (95% CI)
**Overall pooled prevalence**	26	10% (5%, 18%)
**By CKD stage/ definition (n=26)**		
CKD - Stage NR	15	4% (2%, 5%)
CKD Stage 1-3	1	37% (32%, 41%)
CKD Stage 1-5	2	60% (0%, 100%)
CKD Stage >3	7	12% (4%, 23%)
CKD Stage 3-4	1	46% (41%, 52%)
**By SMI (n=28)**		
Bipolar disorder	9	15% (6%, 26%)
Schizophrenia	6	2% (1%, 5%)
Bipolar disorder and schizoaffective disorder	4	12% (2%, 28%)
Mixed SMI	7	13% (0%, 42%)
**By lithium exposure**		
Lithium exposed	6	43% (16%, 73%)
Mixed exposure	8	7% (3%, 11%)
Not reported	12	3% (2%, 5%)
**By setting**		
Population	14	4% (3%, 6%)
Community	4	17% (3%, 39%)
Inpatient	3	28% (0%, 97%)
Inpatient and community	1	27% (20%, 34%)
Not reported	4	7% (2%, 27%)

**By year of publication (n=26)**		
2006-2010	2	12% (0%, 60%)
2011-2015	6	6% (2%, 11%)
2016-2020	5	10% (1%, 26%)
2021-2025	13	12% (3%, 28%)

Figure S1. Pooled prevalence of CKD among people with SMI including Boivin et al. 2023

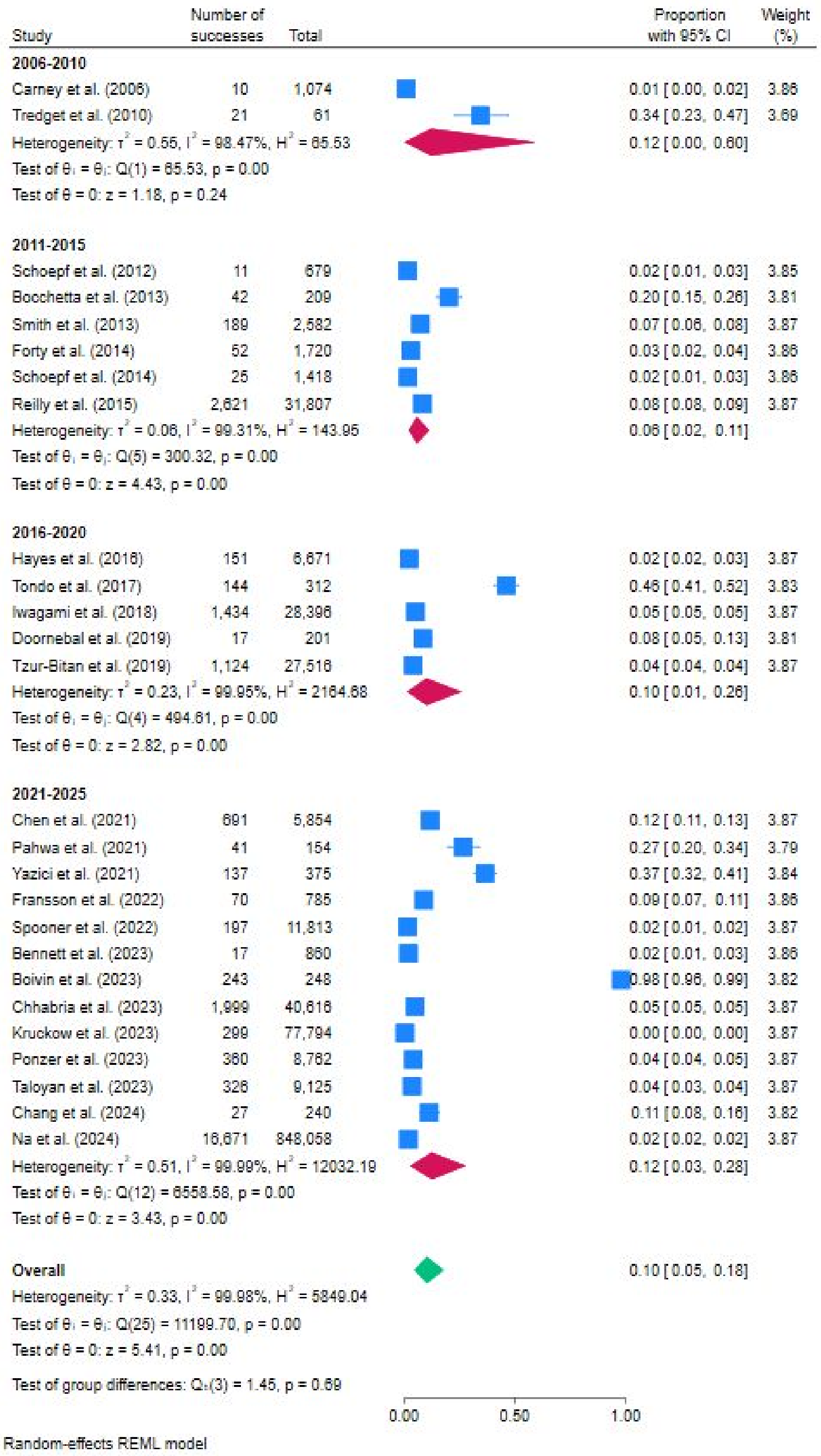

Supplementary Material 3 – Funnel plots

Figure S2 Funnel plot including Boivin et al. (56)

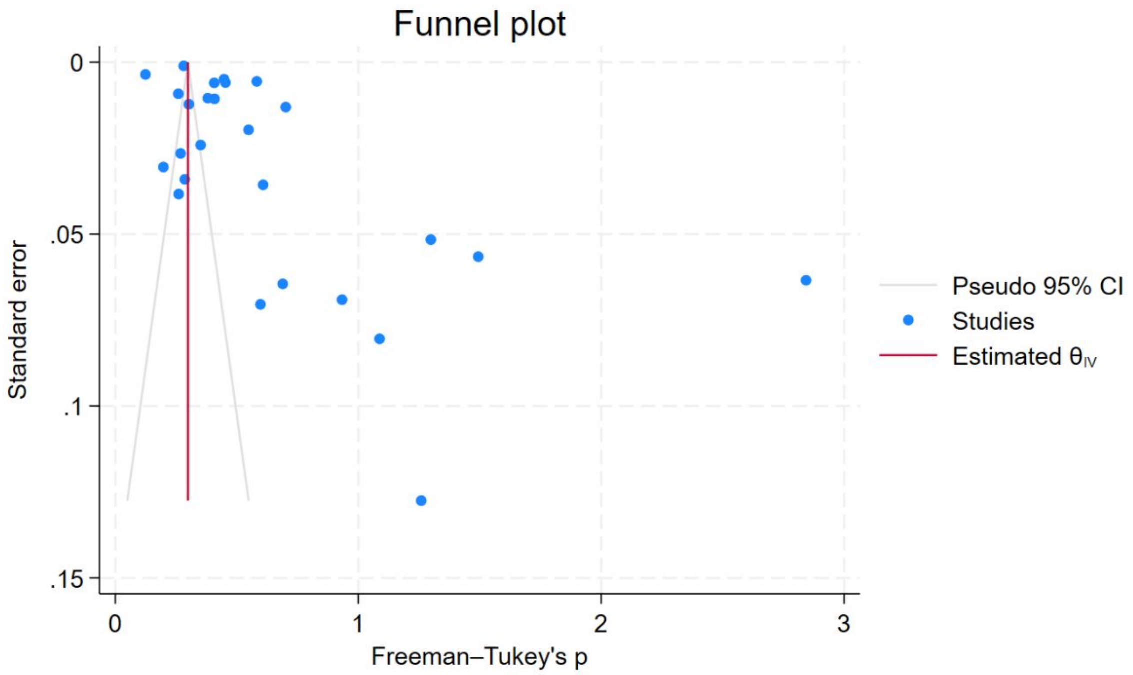

Figure S3 Funnel plot excluding Boivin et al. (56)

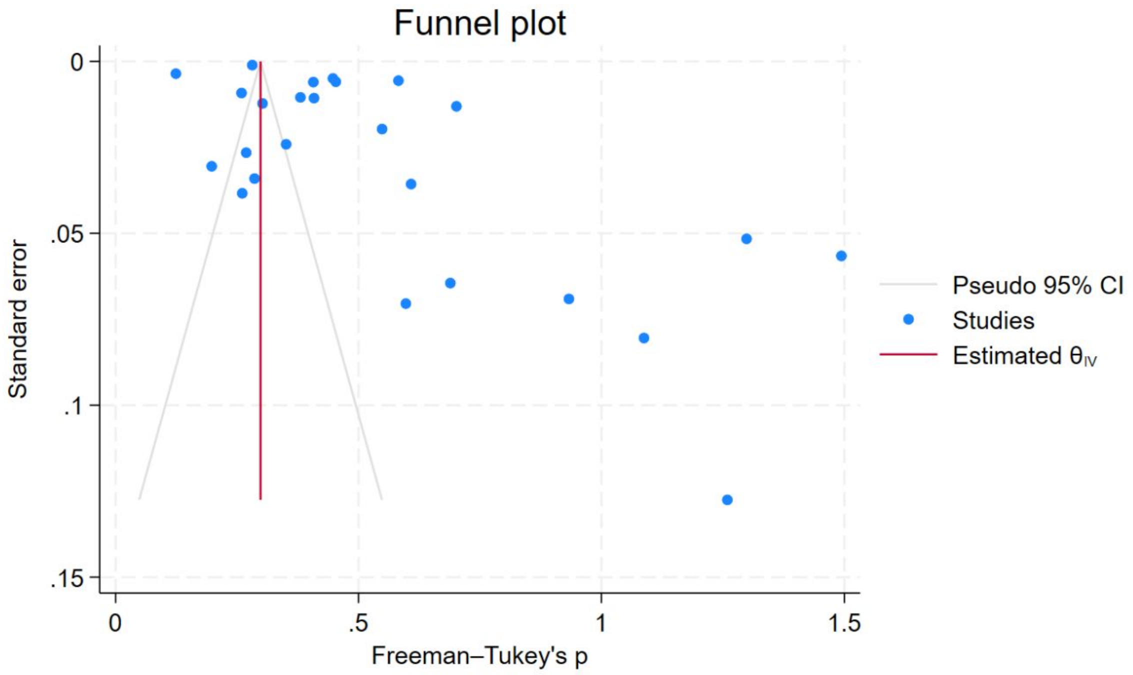

Supplement: Supplementary Material [file EMS213308-supplement-Supplementary_Material.pdf]
